# Effectiveness and Safety of GLP-1 Receptor Agonists in Patients with Type 1 Diabetes

**DOI:** 10.3390/jcm13216532

**Published:** 2024-10-30

**Authors:** Sumaya N. Almohareb, Osamah M. Alfayez, Shoroq S. Aljuaid, Walaa A. Alshahrani, Ghalia Bakhsh, Mohammed K. Alshammari, Majed S. Al Yami, Omar A. Alshaya, Abdullah S. Alomran, Ghazwa B. Korayem, Omar A. Almohammed

**Affiliations:** 1 Department of Pharmacy Practice, College of Pharmacy, King Saud bin Abdulaziz University for Health Sciences, Riyadh 11481, Saudi Arabia; salmohareb1@gmail.com (S.N.A.); omaraalshaya@gmail.com (O.A.A.); 2 King Abdullah International Medical Research Center, Riyadh 11481, Saudi Arabia; alshahraniiwa@gmail.com; 3 Pharmaceutical Care Department, King Abdulaziz Medical City, Riyadh 11481, Saudi Arabia; 4 Department of Pharmacy Practice, College of Pharmacy, Qassim University, Qassim 52571, Saudi Arabia; oalfayez@qu.edu.sa; 5 Department of Pharmaceutical Care, Prince Sultan Military Medical City, Riyadh 12233, Saudi Arabia; shoroqaljuaid@gmail.com (S.S.A.); aalomran@outlook.com (A.S.A.); 6 Department of Pharmaceutical Care, King Fahad Medical City, Riyadh 12231, Saudi Arabia; gh.bakhsh@gmail.com (G.B.); ii.kanan101@gmail.com (M.K.A.); 7 Department of Pharmacy Practice, College of Pharmacy, Princess Nourah Bint Abdulrahman University, Riyadh 11671, Saudi Arabia; ghazwa.krayem@gmail.com; 8 Department of Clinical Pharmacy, College of Pharmacy, King Saud University, Riyadh 11451, Saudi Arabia; 9 Pharmacoeconomics Research Unit, College of Pharmacy, King Saud University, Riyadh 11451, Saudi Arabia

**Keywords:** diabetes mellitus type 1, glucagon-like peptide-1 receptor agonist, treatment outcome, safety, body mass index, body weight

## Abstract

**Background**: GLP-1 receptor agonists (GLP-1RA) are used in the management of type II diabetes mellitus or obesity, although its role in patients with type I diabetes mellitus (T1DM) has been debated. This study aimed to investigate the efficacy and safety of GLP-1RA in patients with T1DM using real-world data. **Methods**: This multicenter, retrospective study was conducted at three tertiary medical centers in Riyadh, Saudi Arabia. The study followed up patients (>16 years old) with T1DM treated with insulin followed by GLP-1RA add-on therapy. The efficacy outcomes included changes in HbA1c, body weight, and insulin requirements from baseline to each follow-up visit. The main safety outcomes assessed included hypoglycemic events and gastrointestinal (GI) adverse events. **Results**: The study included 144 patients with a mean age of 33.0 ± 10.1 years. Semaglutide was the most used GLP-1RA (63.9%) followed by liraglutide (34.0%). From baseline to 3-month follow-up, HbA1c (mean difference (MD) = −0.8%; *p* = 0.0053), weight (MD = −2.4 kg; *p* = 0.0253), and daily basal insulin dose (MD = −2.1 units; *p* = 0.0349) were significantly reduced. Likewise, HbA1c (MD = −0.5%; *p* = 0.0004), weight (MD = −3.6 kg; *p* < 0.0001), and daily basal insulin (MD = −2.4 units; *p* = 0.0282) were significantly reduced at the 4–6-month follow-up. The significant reductions in HbA1c, weight, and daily basal insulin levels were consistent for up to 18-month follow-up. Only one patient had a major hypoglycemic event, whereas 8.3% of the patients had GI adverse events. **Conclusions**: Overall, significant improvements in glycemic control, weight loss, and insulin requirements were observed with the use of GLP-1RA in patients with T1DM, with a limited number of GI adverse events.

## 1. Introduction

Type 1 diabetes mellitus (T1DM) is a chronic autoimmune disease characterized by hyperglycemia and is mainly attributed to insulin deficiency, which occurs as a result of the loss of pancreatic islet *β*-cells. Before the introduction of insulin therapy in 1922, T1DM was a fatal illness [1]. Since *β*-cell activity is either completely or almost completely lacking in people with T1DM, insulin administration is crucial [2]. However, insulin therapy regimens are complex, with basal insulin used to control glucose overnight and between meals, bolus doses to control glucose at mealtime, and correction doses if needed for hyperglycemia. Insulin therapy is affected by behavioral habits and eating patterns; thus, patients need to adjust their insulin doses frequently. Many patients with T1DM fail to meet the glycemic goals required to either prevent or delay the progression of diabetic complications, which persistently imposes a considerable clinical and psychological burden [3]. In addition, insulin therapy is associated with several side effects such as hypoglycemia and weight gain, which may lead some patients to lower their insulin dose [4]. The limitations and challenges of the current insulin therapy have prompted the investigation of alternative treatment options for patients with T1DM.

Glucagon-like peptide-1 (GLP-1) is a multifaceted hormone with diverse pharmacological effects. GLP-1 has numerous metabolic effects, including glucose-dependent stimulation of insulin secretion, decreased gastric emptying, inhibition of food intake, increased natriuresis and diuresis, and modulation of rodent *β*-cell proliferation [5]. GLP-1 receptor agonists (GLP-1RA) are currently used in clinical practice for the treatment of type-2 diabetes mellitus (T2DM) and obesity [5,6].

Emerging but conflicting evidence exists regarding the use of GLP-1RA in patients with T1DM. A randomized open-label clinical trial including 18 patients with T1DM showed that the addition of exenatide reduced insulin requirements in patients with new-onset disease, resulting in significant weight loss with no hypoglycemic risk [7]. Two other clinical trials showed that exenatide as an add-on to standard insulin therapy in patients with T1DM did not significantly reduce HbA1c, glycemic excursions, or glycemic variability compared with placebo when used either as a short-acting drug three times daily or as an extended release once weekly [8,9]. In addition, two large randomized clinical trials (RCTs) of GLP-1RA in patients with T1DM were conducted to evaluate the use of liraglutide 1.8 mg daily compared to placebo as an add-on to standard insulin therapy. The ADJUNCT ONE trial was performed over 52 weeks and enrolled 1398 patients, whereas the ADJUNCT TWO trial was conducted over 26 weeks and enlisted 835 patients. Both studies showed a significant reduction in HbA1c levels, ranging between 0.33% and 0.54%, and body weight, with an average of approximately 5 kg, as well as a lower insulin requirement. However, these positive outcomes were associated with higher rates of hypoglycemic events with liraglutide 1.2 mg and hyperglycemia with ketosis for liraglutide 1.8 mg [10,11]. Another clinical trial including 44 overweight or obese adults with T1DM found that adding liraglutide as an add-on therapy to insulin was associated with a reduction in HbA1c from baseline by 0.5%, total daily insulin by 16%, and mean body weight by 6.3 kg compared with a placebo group. Additionally, the time spent in the glycemic target range increased, whereas the risk of hypoglycemia did not differ between the groups at the end of treatment [12]. Furthermore, liraglutide was evaluated in a systematic review and meta-analysis (MA) that included five clinical trials involving 2445 patients with T1DM. The results demonstrated that the addition of liraglutide improved glycemic control, reduced the total daily insulin dose, and induced significant weight loss with a modest HbA1c reduction. However, gastrointestinal (GI) adverse effects were significantly higher in the liraglutide group than those in the placebo group [13].

The number of published retrospective studies based on real-world data is limited [14,15,16,17,18,19]. All these studies have demonstrated a reduction in HbA1c ranging from −0.4% to −0.7%. Weight was significantly reduced in two of these four studies, and none of them showed a significant increase in diabetic ketoacidosis (DKA) or severe hypoglycemic events, which is inconsistent with the findings of previous RCTs. Although there is some potential for GLP-1RA in patients with T1DM, the evidence is still limited, and more clinical trials and real-world evidence are needed before deciding on its role in these patients. In the present study, we investigated the efficacy and safety of GLP-1RA in Saudi patients with T1DM using real-world data.

## 2. Materials and Methods

### 2.1. Study Design, Setting and Patients

A multicenter retrospective study was conducted, including patients from three tertiary medical centers in Riyadh, Saudi Arabia: King Abdulaziz Medical City (KAMC), King Fahad Medical City (KFMC), and Prince Sultan Military Medical City (PSMMC). The study included patients older than 16 years with a confirmed diagnosis of T1DM, as documented in the physicians’ progress note, who were treated with insulin and started on GLP-1RA as an add-on therapy for better management of diabetes or weight loss. The study excluded patients with T2DM or other types of diabetes and those who did not have a documented baseline lab or follow-up visit after starting GLP-1RA. This study was approved by the Institutional Review Boards (IRBs) at three study sites: KAMC (ref. no. NRC22R/565/11), KFMC (ref. no. 22-627), and PSMMC (ref. no. HP-01-R079).

Data were collected from patients administered these medications between January 2016 and December 2023. Patients were followed up until GLP-1RA medication was stopped, swapped for another GLP-1RA, or follow-up for 18 months was reached. To assess treatment outcomes, patients had to have at least one follow-up visit after starting GLP-1RA and continue using it for at least one month.

### 2.2. Data Collection

The collected data included patient demographics (age, sex, weight, and body mass index [BMI]) and medical history (diabetes duration, comorbid conditions, and diabetes complications) at baseline and each follow-up visit for weight and BMI. In addition, treatment-related information was collected, including the name and initial and maintenance doses of GLP-1RA, insulin type (basal or prandial), documented total daily doses (TDDs) used from each type at baseline and at each follow-up visit, and concomitant medications used at baseline. Vital signs (systolic and diastolic blood pressure [BP]) and laboratory values (HbA1c, fasting blood glucose [FBG], serum creatinine, creatinine clearance [CrCl], estimated glomerular filtration rate [eGFR], blood urea nitrogen [BUN], albumin, albumin-to-creatinine ratio, potassium, hemoglobin, and lipid profile) were collected at baseline and at each follow-up visit. The incidence of GI events (i.e., nausea, vomiting, diarrhea, or constipation) reported by patients, diabetic ketoacidosis (DKA), minor (i.e., events that did not require emergency department visits or hospitalization) or major (events that required emergency department visits or hospitalization) hypoglycemic events, volume depletion, and hospitalization during therapy were also collected, in addition to the date of discontinuation of GLP-1RA, if it occurred, and the reason for discontinuation of therapy, if documented.

### 2.3. Outcome Measures

The main efficacy outcomes were defined as changes in glycemic control (represented by HbA1c), body weight, and insulin requirements from baseline to follow-up. Safety outcomes were defined as minor or major hypoglycemic events, DKA, and adverse drug reactions. Other efficacy outcomes included changes in systolic and diastolic BP, FBG, and lipid profiles (i.e., total cholesterol, low-density lipoprotein [LDL], high-density lipoprotein [HDL], and triglycerides). Patient efficacy outcomes were assessed at different time intervals up to 18 months of follow-up (within 3 months and between 3 and 6, 6 and 9, 9 and 12, and 12 and 18 months) and compared in each interval to the patients’ baseline levels for these variables.

### 2.4. Statistical Analysis

Descriptive statistics, mean with standard deviation (mean ± SD) for continuous data, and frequency with percentage for categorical data, were used to describe patients’ baseline characteristics and outcomes. Changes in patient efficacy outcomes from baseline to each follow-up interval were assessed using paired *t*-tests. Comparisons with *p*-value < 0.05 were considered statistically significant. Data were extracted from the patients’ electronic medical records and entered into the research electronic data capture system (REDcap, Vanderbilt University, Nashville, TN, USA). Data were managed using Microsoft Excel version 2019 (Microsoft Corp., Redmond, WA, USA) and all statistical analyses were performed using the SAS software, version 9.4 (SAS Institute Inc., Cary, NC, USA).

## 3. Results

### 3.1. Patients’ Baseline Characteristics

A total of 144 patients diagnosed with T1DM who were receiving any GLP-1RA were included in the analysis. The mean age for the patients was 33.0 ± 10.1 years with a mean duration for T1DM of 16.5 ± 7.8 years, and 64.6% of them were female. The mean weight in these patients was 89.9 ± 16.3 kg with an average BMI of 34.0 ± 5.7 kg/m^2^. Dyslipidemia was the most common comorbidity, affecting 38.2% of patients, followed by hypothyroidism in 19.4%, and none of our patients had thyroid cancer in the past. The most prevalent historical diabetes-related complication was DKA, which historically occurred in 19.4% of patients. In addition to insulin, statins were the most common medications used by 39.6% of patients, whereas 13.9% used metformin as an add-on therapy before starting GLP-1RA.

Most of our patients were administered multiple insulin injections (92.4%), although 7.6% used insulin pumps. Among those receiving multiple injections, all were on basal insulin and the majority were on prandial insulin with basal insulin (97.7%). The mean TDD of insulin was 81.2 ± 33.2 units/day (0.9 units/kg/day), with an average of 33.2 ± 14.2 units of basal insulin and 48.1 ± 23.1 units of bolus insulin.

Generally, our patients had an overall normal mean BP, with an average systolic and diastolic BP of 124.5 ± 14.3 and 72.9 ± 11.3 mmHg, respectively. The mean HbA1c level among all patients included in the study at baseline was 8.9 ± 1.6%, whereas the mean FBG level was 214.5 ± 88.8 mg/dL. Our patients, on average, had a high LDL level (125.1 ± 40.6 mg/dL) while maintaining normal levels of overall cholesterol, HDL, and triglycerides. The patients in the study presented good renal functions as determined by the renal parameter assessments, with an average serum creatinine level and eGFR of 0.7 ± 0.2 mg/dL and 103.3 ± 19.2 mL/min/1.73 m^2^, respectively. Table 1 summarizes the patients’ baseline characteristics, medications used, vital signs, and laboratory values at baseline for all included patients.

### 3.2. Study GLP-1RA

Among the 144 patients, the majority (92 patients; 63.9%) were on semaglutide, and most of them started on the recommended 0.25 mg once weekly dose (88.0%) and continued the 1 mg once weekly injections as a maintenance dose (80.4%). Of the 49 patients (34.0%) who took liraglutide, most (83.7%) began with a starting dose of 0.6 mg once daily, and 87.8% continued on the maintenance dose of 1.8 mg once daily. Only three patients were on dulaglutide therapy.

Approximately one-fourth of the patients treated with semaglutide decided to interrupt therapy (21/92 patients; 22.8%) before the end of the 18-month follow-up period, with an average treatment duration of 7.9 ± 3.7 months. However, more than half of those treated with liraglutide (28/49 patients; 57.1%) experienced therapy interruption before the end of the study follow-up period, with an average duration of 9.2 ± 4.2 months. Notably, liraglutide was available in the hospital first but was removed from the hospital formulary during the study follow-up period. In terms of the reasons for discontinuation of therapy, four patients mentioned that being pregnant or planning to become pregnant was the reason for discontinuing therapy. In the semaglutide group, three patients discontinued their medication due to GI side effects, which was the most frequently cited cause for discontinuation of therapy among these patients. Due to the removal of liraglutide from some hospitals’ formularies, two patients discontinued the drug, and eight switched to another GLP-1RA. In addition, COVID-19 caused five patients to be lost to follow-up, preventing them from accessing the medication, and two patients experienced GI side effects or intolerance. Table 2 summarizes the types of GLP-1RA used with doses, duration of therapy (for patients who discontinued treatment), and reasons for discontinuation.

### 3.3. Efficacy Outcomes

#### 3.3.1. Changes in HbA1c, Body Weight, Daily Insulin, and Lipids During 3-Month Follow-Up

For patients with follow-up ≤ 3 months (*n* = 31 patients), the HbA1c declined from 9.3 ± 2.0% at baseline to 8.8 ± 1.8% within three months revealing a significant reduction of 0.8 ± 1.2% (*p* = 0.0053). Additionally, the mean reduction in weight and BMI were 2.4 ± 5.2 kg (*p* = 0.0253) and 0.9 ± 2.1 kg/m^2^ (*p* = 0.0332), respectively. Moreover, the daily basal insulin dose declined from 32.0 to 29.8 units with a mean significant reduction of 2.1 ± 5.2 units (*p* = 0.0349). All comparisons of efficacy outcomes are presented in Table 3 and Table 4.

#### 3.3.2. Change in HbA1c, Weight, Daily Insulin, and Lipids at 4–6-Month Follow-Up

For patients with a follow-up within 4–6 months of GLP-1RA initiation (*n* = 74 patients), the HbA1c declined from 9.0 ± 1.5% at baseline to 8.4 ± 1.3% within 4–6-month follow-up, revealing a significant reduction at 0.5 ± 1.0 (*p* = 0.0004). The mean reductions in weight and BMI were 3.6 ± 5.0 kg (*p* < 0.0001) and 1.4 ± 1.9 kg/m^2^ (*p* < 0.0001), respectively. Likewise, the daily basal insulin dose declined, with a significant mean reduction of −2.4 ± 9.2 units (*p* = 0.0282).

#### 3.3.3. Changes in HbA1c, Body Weight, Daily Insulin, and Lipids at 7–9-Month Follow-Up

For patients with a follow-up within 7–9 months of GLP-1RA initiation (*n* = 54 patients), the HbA1c declined from 8.9 ± 1.7% at baseline to 8.3 ± 1.6% within 7–9-month follow-up, revealing a significant reduction of 0.6 ± 1.2 (*p* = 0.0004). The mean reductions in weight and BMI were 6.0 ± 7.7 kg (*p* < 0.0001) and 2.3 ± 3.0 kg/m^2^ (*p* < 0.0001), respectively. The TDD of insulin declined from 0.9 ± 0.3 units/kg/day at baseline to 0.8 ± 0.5 units/kg/day with a significant mean reduction of 0.1 ± 0.4 units/kg/day (*p* = 0.0408). In addition, a significant reduction in LDL and triglyceride was observed from baseline to 7–9-month with a mean reduction of −16.7 ± 39 mg/dL (*p* = 0.0259) and −24.1 ± 52 mg/dL (*p* = 0.0216), respectively.

#### 3.3.4. Changes in HbA1c, Body Weight, Daily Insulin, and Lipids at 9–12- and 12–18-Month Follow-Ups

For patients with a follow-up within 9–12 months (*n* = 37 patients) or 12–18 months (*n* = 38 patients) of GLP-1RA initiation, the HbA1c declined from baseline with a significant mean reduction of 0.5 ± 1.2% (*p* = 0.0210) and 0.5 ± 0.8% (*p* = 0.0022). Additionally, the mean reduction in weight (and BMI) from baseline to 9–12- and 12–18-month follow-ups was significant at 5.6 kg (2.1 kg/m^2^) and 5.2 kg (1.9 kg/m^2^), respectively. Moreover, the daily basal insulin dose significantly declined from baseline to 12–18-month follow-up by a mean reduction of 4.1 units ± 9.2 units (*p* = 0.0101).

### 3.4. Safety Outcomes

Minor hypoglycemic events were reported by 18 patients (12.5%), and the total number of these events was 55 during the 18-month follow-up period. Major hypoglycemic events were observed in only one patient during all follow-up periods. GI events were the most prevalent adverse events reported by the patients and were reported in 12 patients (8.3%). Hospitalization or ED visits for any reason during therapy were observed in 21 (14.6%) patients, most of whom were admitted once during the follow-up period, as shown in Table 5.

## 4. Discussion

Although there have been significant technological and insulin delivery improvements in recent decades [20], a significant proportion of adults with T1DM cannot achieve the target HbA1c levels [21,22]. Efforts to identify new and effective treatments for T1DM have been unsuccessful. Moreover, studies have reported that a proportion of patients with T1DM still utilize supplementary (off-label) glucose-lowering therapy with insulin [16,23]. In this observational study of patients with T1DM, the use of GLP-1RA, mostly semaglutide or liraglutide, as an add-on therapy to insulin resulted in a statistically significant reduction in HbA1c level, body weight, BMI, and basal insulin dose. In terms of safety, GLP-1RA did not result in a higher occurrence of severe hypoglycemia or DKA, whereas, as anticipated, GI events were the most reported adverse events.

In our study, GLP-1RA resulted in a reduction in HbA1c that ranged from −0.5% to −0.8%, depending on the follow-up period, which is higher than what has been previously reported in RCTs. Taking into consideration the differences in study design, our study reported the change in HbA1c from baseline to follow-up without comparison with the control group. Liraglutide is the most studied GLP-1RA used in patients with T1DM. The largest RCTs to date have shown statistically significant reductions in HbA1c levels when liraglutide is combined with insulin therapy, mostly in a dose-dependent manner [10,11]. For instance, in the ADJUNCT ONE trial, the HbA1c reduction was −0.2% (95%CI, −0.32 to −0.07) and −0.15% (95%CI, −0.21 to −0.03%) for liraglutide 1.8 mg and 1.2 mg, respectively [10]. In a recent MA that included 11 RCTs, of which eight assessed liraglutide, two assessed exenatide, and one assessed albiglutide, and the overall reduction in HbA1c with GLP-1RA was −0.21% (95%CI −0.33 to −0.10). HbA1c reduction was mostly driven by liraglutide-based studies; when examining liraglutide alone, the mean reduction in HbA1c was modest at −0.26% (95%CI −0.26 to −0.14) [3].

To date, no RCTs have provided information on whether the reduction in HbA1c levels would continue beyond one year of treatment, which is crucial given the chronic nature of T1DM. In our study, HbA1c reduction remained statistically significant at the 18-month follow-up, which is consistent with the results of studies using real-world data [15,16,17,18,19]. For instance, a study that included 54 participants with a follow-up duration of up to 2 years showed a statistically significant reduction in HbA1c levels of −0.7% (*p* = 0.0018). Most patients were on long-acting GLP-1RA, i.e., as semaglutide (63%) or dulaglutide (35%) [15]. The largest real-world study by Anson et al. included 933 patients with T1DM receiving GLP-1RA after propensity score matching. In their study, liraglutide was administered to 37% of the patients, semaglutide to 25%, dulaglutide to 24%, and exenatide to 13%. Importantly, the mean change in HbA1c over 5-year follow-up was −0.5% [16]. A recent observational study by Garg and colleagues investigated the use of semaglutide in 50 overweight or obese patients with T1DM. They observed a statistically significant decline in mean HbA1c (−0.66%) at 1-year follow-up with semaglutide compared to the control group [18]. Similarly, the only observational study that evaluated tirzepatide reported a sustained HbA1c reduction of −0.67% at 1-year follow-up [19]. The differences in GLP-1RA used in these studies, as well as differences in study design, may have contributed to the observed differences in HbA1c reduction. Interestingly, some studies have reported that patients with positive C-peptide levels tend to have better GLP-1RA efficacy and safety [10,11], which should be further investigated. However, this usage is still considered off-label, and further data are required to definitively establish the safety of this combination.

The occurrence of insulin side effects, such as weight gain, can have a negative impact on patient adherence to insulin treatment. Moreover, increased body weight is a recognized risk factor of cardiovascular disease [24], which is a well-established cause of early mortality in individuals with T1DM [25,26]. Therefore, the use of medications that can reduce weight, in combination with insulin, to offset weight gain is a reasonable approach for obese patients with T1DM. GLP-1RA drugs are known to increase satiety and decrease food intake and have been proven to decrease weight in obese patients with or without diabetes [2]. However, the effect of GLP-1RA on body weight is not the same across all medications [20].

In patients with T1DM, the most recent MA found that GLP-1RA resulted in statistically significant weight loss in patients with T1DM. The pooled analysis showed a mean reduction of −4.04 kg (95%CI, −4.8 to −3.27). However, this reduction was only observed with liraglutide (−3.98 kg) and exenatide daily injection (−8.3 kg) [3]. Notably, the MA did not include studies on semaglutide, dulaglutide, or tirzepatide, which are known to have a better effect on weight reduction. In our study, the mean reduction in bodyweight from baseline ranged from −2.4 kg to −6.0 kg at different follow-up periods. Similarly, the statistically significant reduction in BMI ranged from −0.9 to −2.3 kg/m^2^.

Moreover, GLP-1RA is believed to have a more significant impact on weight if the patient weight at baseline was high [3]. Notably, in our study, the mean BMI at baseline (34 ± 5.7 kg/m^2^) was higher than that of the previously published RCTs, which ranged from 22.4 to 31.7 kg/m^2^. Patients in the study by Anson et al. had a baseline BMI of 33.1 ± 6.9 kg/m^2^ [16], which is fairly similar to our study. Moreover, the average weight at baseline was 89.9 kg in our cohort, whereas other observational studies reported average weights ranging from 83 to 103.8 kg [15,16,17,18,19]. All the previously mentioned studies observed a reduction in body weight, with the highest weight reduction observed with tirzepatide (18.5%; >20 kg), except for the study by Anson et al., which reported an average increase of +1.5 kg at the 5-year follow-up. The authors provided an explanation for these results, which included a good number of cohorts on dulaglutide (24%) and exenatide (13%), which might be less effective in reducing weight than semaglutide. Additionally, a significant proportion of patients discontinued GLP-1RA after three years [16].

Overall, weight reduction may have primarily contributed to the reduction in HbA1c levels after GLP-1RA treatment. In contrast, normal-weight patients might exhibit HbA1c reduction from GLP-1RA through other mechanisms, such as inhibition of glucagon secretion. Therefore, further evaluation is required to determine the benefits of long-acting GLP-1RA, such as semaglutide or tirzepatide, in patients with T1DM at normal or subnormal weight.

In this study, the TDD of insulin (units/kg) and prandial insulin did not show a statistically significant reduction across all treatment follow-up periods. However, basal insulin dose demonstrated a statistically significant reduction from baseline, which ranged from −1 unit to −4.1 units of insulin. Unlike our study, in the previously mentioned MA by Tan et al., the use of GLP-1RA showed a reduction in prandial insulin dose, basal insulin dose, and TDD of insulin [3], which is consistent with the ADJUNCT ONE and ADJUNCT TWO trials [10,11]. This was also observed in an observational study by Mohandas et al. [15]. The reason why only basal insulin requirements were reduced in our study is unclear, especially because previous studies have demonstrated a greater effect on prandial insulin [10,11]. This may be because most patients in our cohort were treated with semaglutide.

The effect of adding GLP-1RA on metrics for CGM among patients with T1DM was evaluated in a recent MA of six RCTs that included 378 patients. The results showed no significant benefit on time in range, −0.22% (95%CI, −2.39 to 1.95; *p* = 0.84), with slightly more time below range, 1.13% (95%CI, 0.50 to 1.76; *p* < 0.001), compared to placebo [27]. In our study, only 27 patients were using CGM devices, making it difficult to draw a conclusion from our sample and this was not the aim of the study. However, adding GLP-1RA did increase the time below the target glycemic range during some follow up periods but that was not significant, as previously indicated. Therefore, more research is needed where we assess the impact of using GLP-1RA in patients with T1DM who are using CGM.

The results regarding the safety of GLP-1RA use in T1DM are inconsistent. In the ADJUNCT ONE and ADJUNT TWO trials, the authors concluded that the use of liraglutide in patients with T1DM might be limited owing to an increase in the rates of symptomatic hypoglycemia and hyperglycemia with ketosis [10,11]. Although DKA is expected to occur more frequently in real life than in clinical trials owing to less strict patient selection and monitoring, observational studies have not found a large increase in the incidence of DKA [15,16,17,18,19]. A reduction in insulin dose requirement with GLP-1RA could raise the risk of DKA, which could partially explain the increased hyperglycemia with ketosis events in controlled settings [10,11]. Greater flexibility in insulin dose adjustment in clinical practice might have decreased the chances of such complications. Interestingly, all confirmed episodes of DKA in the ADJUNCT ONE trial occurred among patients with negative C-peptide levels [10]. Nevertheless, patients with T1DM taking GLP-1RA should be regularly monitored for ketones, particularly when experiencing DKA symptoms.

In the aforementioned MA, there was no increase in severe hypoglycemic events among GLP-1RA users. However, an increase in general hypoglycemia events was noted with an odds ratio of 1.89 (95%CI, 1.06 to 3.39) [3]. In real-world studies, the rates of severe hypoglycemia were not higher than those in the general T1DM population [15,16,17,18,19]. Most real-world studies have not reported general hypoglycemic events, mostly due to a lack of documentation. In our study, only one episode of major hypoglycemia was reported, whereas minor hypoglycemia, which did not require any medical attention, was reported by 18 patients (12.5%) during the 18-month follow-up period.

The previously mentioned MA found that GLP-1RA has resulted in a statistically significant increased incidence of GI side effects in patients with T1DM (OR, 2.96; 95%CI, 2.33 to 3.77) [3]. On the other hand, most observational studies did not report data regarding GI side effects. However, Mohandas et al. found 64.8% of participants had GI side effects [15], while Anson et al. did not find a statistically significant increase in these adverse effects [16]. In our study, 12 patients (8.3%) reported GI adverse events, which were the most prevalent adverse events. Overall, the MA by Tan and colleagues did not find an increase in the incidence of severe adverse events in patients with T1DM receiving GLP-1RA, which was also observed in the observational studies published to date.

Overall, the reason for initiating GLP-1RA in addition to insulin in real-world settings is to achieve better glycemic control, induce weight loss, and reduce the insulin dose. Although the HbA1c reduction might seem modest in RCTs and some observational studies, it has been previously reported that a 0.5% reduction in HbA1c is considered clinically significant [28,29,30]. Moreover, given the pathophysiological differences between T1DM and T2DM, it is anticipated that the HbA1c reduction achieved with GLP-1RA will not be at the magnitude seen in T2DM. Although, in the ADJUNCT ONE trial and the aforementioned MA, the average HbA1c reduction was around −0.2% for liraglutide 1.8 mg, the observational studies, on the other hand, reported an average reduction that ranged between −0.4% and −0.67%. Such a reduction in HbA1c might be of greater clinical importance when accompanied by significant and sustainable weight loss and a decrease in insulin dose in overweight and obese patients with T1DM. Moreover, although there is no evidence, the addition of GLP-1RA might aid in the reduction of CVD risk in patients with T1DM. Whether these benefits are more likely to be seen with more potent GLP-1RA associated with more weight loss, such as semaglutide and tirzepatide, remains to be seen, and further prospective studies with prolonged follow-ups are needed to address the clinical impact on various outcomes, including CVD.

The precise timing for initiating this combination therapy during the progression of T1DM remains uncertain. A case series of 10 patients with T1DM who started semaglutide treatment within 3 months of diagnosis showed interesting results. All patients stopped prandial insulin after three months and had an increase in fasting C-peptide levels, whereas seven patients stopped basal insulin within six months [31]. In an RCT, liraglutide was also found to preserve beta-cell function when used early in patients with T1DM [32]. Finally, an ongoing trial of semaglutide in patients with T1DM and insulin resistance may provide additional information in the near future [33].

Finally, this study had some limitations. For instance, this study investigated mean changes from baseline and did not include a control group. This might have led to a greater reduction in HbA1c observed in our study, −0.5% to −0.8%, compared to other RCTs. The lack of a control group might overestimate the effect of GLP-1RA and make it difficult to control for other factors that might have played a role in reducing the HbA1c. Even though our results (HbA1c reduction) did not differ from those of other observational studies, one must interpret them cautiously. In addition, most patients in the cohort were on semaglutide, and only three patients were on dulaglutide. We did not gather data regarding C-peptide levels or a history of hypoglycemia. In our study, a limited number of patients were using CGM devices, making it difficult to draw a significant conclusion regarding the effect of adding GLP-1RA on metrics for CGM among patients with T1DM. Furthermore, the retrospective nature of our study and the multicenter setting limited our control over available data. However, we included only T1DM patients with a confirmed diagnosis based on documentation from their physicians’ progress notes. However, data on family history of diabetes and the beta-cell antibodies were lacking for most patients, so we were not able to include it. The limitations of the retrospective design should be considered when interpreting the results. Nonetheless, most of our findings generally aligned with the findings of previous RCTs and observational studies. Finally, the data were collected from one country, limiting the generalizability of the results to other populations.

## 5. Conclusions

We found that the addition of GLP-1RA to insulin therapy significantly reduced HbA1c levels, body weight, BMI, and basal insulin dose in patients with T1DM during an 18-month follow-up. GLP-1RA did not increase the risk of severe hypoglycemia or DKA; however, GI events were the most frequently reported adverse events. The findings of this study must be interpreted considering its limitations, such as the absence of a control group. Moreover, a long-term evaluation of the safety of GLP-1RA, the ideal time for starting these medications, and which subgroups of patients with T1DM are likely to benefit is required to guide their use in real-world settings.

## Figures and Tables

**Table 1:** Baseline characteristics of patients on GLP-1 RAs (*n* = 144).

Variable	All
Age, years	33.0 ± 10.1
Gender	
Male	51 (35.4)
Female	93 (64.6)
Weight, kg	89.9 ± 16.3
BMI, kg/m^2^	34.0 ± 5.7
Diabetes duration, years	16.5 ± 7.8
Comorbidities	
Dyslipidemia	55 (38.2)
Hypothyroidism	28 (19.4)
Hypertension	17 (11.8)
Chronic kidney disease	3 (2.1)
Heart failure	1 (0.7)
History of pancreatitis	1 (0.7)
Other comorbidities	20 (13.9)
Diabetes-related complications	
DKA	28 (19.4)
Diabetic retinopathy	7 (4.9)
Diabetic nephropathy	6 (4.2)
Diabetic peripheral neuropathy	1 (0.7)
Cardiovascular disease	1 (0.7)
Cerebrovascular diseases	1 (0.7)
Diabetic foot injury	1 (0.7)
Insulin treatment	
Multiple insulin injections	133 (92.4)
Basal Insulin	133 (100.0)
Prandial Insulin	130 (97.7)
Insulin Pump	11 (7.6)
Total basal insulin, units/day	33.2 ± 14.2
Total bolus insulin, units/day	48.1 ± 23.1
TDD of insulin, units/day	81.2 ± 33.2
TDD of insulin, units/kg/day	0.9 ± 0.4
Other medications	
Statins	57 (39.6)
Metformin	20 (13.9)
ACEI	13 (9.0)
ARBs	9 (6.3)
Aspirin	8 (5.6)
CCBs	6 (4.2)
Ezetimibe	4 (2.8)
Beta-blockers (e.g., bisoprolol, carvedilol)	3 (2.1)
Thiazide diuretics	1 (0.7)
Loop diuretics	1 (0.7)
Blood pressure	
Systolic, mmHg	124.5 ± 14.3
Diastolic, mmHg	72.9 ± 11.3
Laboratory values	
HbA1c, %	8.9 ± 1.6
FBG, mg/dL	214.5 ± 88.8
Use of continuous glucose monitoring (CGM)	27 (18.8)
Time in Range (TIR), %	49.7 ± 14.9
Time above range (TAR), %	38.0 ± 13.6
Time below range (TBR), %	3.7 ± 3.6
Lipid Profile	
Total cholesterol, mg/dL	189.5 ± 42.1
LDL, mg/dL	125.1 ± 40.6
HDL, mg/dL	53.2 ± 15.9
Triglyceride, mg/dL	111.5 ± 58.7
Serum creatinine, mg/dl	0.7 ± 0.2
Creatinine clearance, mL/min	136.7 ± 39.6
eGFR, mL/min/1.73 m^2^	103.3 ± 19.2
BUN, mmol/L	4.0 ± 1.5
Albumin, g/L	41.2 ± 4.7
Albumin-to-creatinine ratio, mg/g	2.2 ± 2.3
Potassium, mmol/L	4.5 ± 0.5
Hemoglobin, g/L	133.4 ± 18.6

Numbers are presented as frequency (%) or mean ± SD. Abbreviations: BMI: body mass index; DKA: diabetic ketoacidosis; TDD: total daily dose; ACEI: angiotensin-converting enzyme inhibitors; ARBs: angiotensin receptor blockers; CCBs: calcium channel blockers; FBG: fasting blood glucose; eGFR: estimated glomerular filtration rate; BUN: blood urea nitrogen; LDL: low-density lipoprotein; HDL: high-density lipoprotein; SD: standard deviation.

**Table 2:** GLP-1RA dosing, discontinuation, duration, and reasons for discontinuation.

	Semaglutide	Liraglutide	Dulaglutide
Semaglutide	92 (63.9)		
Starting dose			
0.25 mg once weekly	81 (88.0)	---	---
0.5 mg once weekly	9 (9.8)	---	---
1.0 mg once weekly	2 (2.2)	---	---
Maintenance dose			
0.25 mg once weekly	2 (2.2)	---	---
0.5 mg once weekly	15 (16.3)	---	---
1.0 mg once weekly	74 (80.4)	---	---
1.5 mg once weekly	1 (1.1)	---	---
Liraglutide		49 (34.0)	
Starting dose			
0.6 mg once daily	---	41 (83.7)	---
1.2 mg once daily	---	1 (2.0)	---
1.8 mg once daily	---	7 (14.3)	---
Maintenance dose			
1.2 mg once daily	---	2 (4.1)	---
1.8 mg once daily	---	43 (87.8)	---
3.0 mg once daily	---	4 (8.2)	---
Dulaglutide			3 (2.1)
Starting dose			
0.75 mg once weekly	---	---	2 (66.7)
1.5 mg once weekly	---	---	1 (33.3)
Maintenance dose			
1.5 mg once weekly	---	---	3 (100.0)
Discontinuation of therapy	21 (22.8)	28 (57.1)	0 (0.0)
Time on GLP-1RA until discontinuation, months	7.9 ± 3.7	9.2 ± 4.2	---
Documented reasons of discontinuation			
Medication removed from hospital formulary	0 (0.0)	2 (4.1)	0 (0.0)
Lost follow-up due to COVID-19	0 (0.0)	5 (10.2)	0 (0.0)
Switched to another GLP-1RA	1 (1.1)	8 (16.4)	0 (0.0)
Gastrointestinal side effects	3 (3.3)	1 (2.0)	0 (0.0)
Pregnancy	2 (2.2)	2 (4.1)	0 (0.0)
Intolerance	1 (1.1)	1 (2.0)	0 (0.0)
Non-compliance	1 (1.1)	2 (4.1)	0 (0.0)

Numbers are presented as frequency (%) or mean ± SD. Gastrointestinal events: nausea, vomiting, or diarrhea. Abbreviations: GLP-1RA: glucagon-like peptide-1 receptor agonist; SD: standard deviation.

**Table 3:** Efficacy outcomes at 3, 4-6, and 7-9 months (Participants’ parameters at baseline and after using GLP-1RA).

Parameters	Up to 3 Months (*n* = 31)				4–6 Months (*n* = 74)				7–9 Months (*n* = 54)			
	Baseline	Follow-Up	MD	*p*-Value	Baseline	Follow-Up	MD	*p*-Value	Baseline	Follow-Up	MD	*p*-Value
Weight, kg	84.3 ± 17.0	81.0 ± 19.5	−2.4 ± 5.2	**0.0253**	91.0 ± 17.7	86.9 ± 17.2	−3.6 ± 5.0	**<0.0001**	90.6 ± 15.6	83.3 ± 16.1	−6.0 ± 7.7	**<0.0001**
BMI, kg/m^2^	32.6 ± 6.0	31.3 ± 7.0	−0.9 ± 2.1	**0.0332**	34.2 ± 6.4	33.0 ± 6.5	−1.4 ± 1.9	**<0.0001**	34.2 ± 5.4	31.8 ± 6.1	−2.3 ± 3.0	**<0.0001**
TDD of insulin, units/day	77.1 ± 33.5	75.5 ± 32.0	−1.3 ± 14.7	0.6471	81.5 ± 33.1	77.2 ± 34.7	−4.3 ± 21	0.0846	84.5 ± 32.9	81.7 ± 36.7	−4.0 ± 18.6	0.1383
Basal Insulin	32.0 ± 14.0	29.8 ± 13.8	−2.1 ± 5.2	**0.0349**	34.8 ± 13.7	32.4 ± 14.1	−2.4 ± 9.2	**0.0282**	32.7 ± 14.9	31.6 ± 16.0	−1.0 ± 5.7	0.2104
Prandial insulin	45.1 ± 24.0	45.7 ± 21.9	0.8 ± 14.0	0.7461	46.6 ± 23.0	44.7 ± 25.0	−1.9 ± 18	0.3673	51.9 ± 21.6	49.9 ± 24.6	−2.9 ± 15.1	01781
TDD of insulin, units/kg/day	0.9 ± 0.3	0.8 ± 0.4	−0.1 ± 0.3	0.1963	0.9 ± 0.4	0.8 ± 0.4	−0.1 ± 0.4	**0.0115**	0.9 ± 0.3	0.8 ± 0.5	−0.1 ± 0.4	**0.0408**
Time in Range (TIR), %	43.7 ± 15.8	52.6 ± 25.6	17.0 ± 38.0	0.5195	51.3 ± 20.2	51.3 ± 15.8	2.8 ± 12.2	0.6347	51.6 ± 12.2	50.3 ± 18.1	−3.5 ± 16.5	0.7004
Time Above Range (TAR), %	37.6 ± 13.9	31.1 ± 23.5	−1.7 ± 18.5	0.8907	32.0 ± 8.5	40.6 ± 20.0	4.2 ± 14.6	0.5556	44.6 ± 13.5	41.8 ± 19.7	−4.8 ± 17.5	0.6257
Time Below Range (TBR), %	4.0 ± 4.7	2.2 ± 3.1	−0.7 ± 1.2	0.4226	3.8 ± 3.4	6.3 ± 7.9	0.4 ± 0.9	0.3739	2.7 ± 3.0	6.3 ± 11.0	0.5 ± 1.0	0.3910
Vital signs												
Systolic BP, mmHg	125.9 ± 19.4	124.2 ± 19.9	2.9 ± 15.1	0.2978	125.6 ± 14.0	122.5 ± 14.5	−2.1 ± 15.1	0.2597	122.9 ± 13.1	121.8 ± 14.4	0.0 ± 13.6	1.0000
Diastolic BP, mmHg	73.4 ± 14.7	71.3 ± 11.8	−2.9 ± 11.1	0.1772	74.4 ± 11.7	73.5 ± 11.3	−0.1 ± 12.3	0.9305	72.1 ± 11.0	72.9 ± 12.0	0.5 ± 10.2	0.7372
Laboratory values												
HbA1c, %	9.3 ± 2.0	8.8 ± 1.8	−0.8 ± 1.2	**0.0053**	9.0 ± 1.5	8.4 ± 1.3	−0.5 ± 1.0	**0.0004**	8.9 ± 1.7	8.3 ± 1.6	−0.6 ± 1.2	**0.0020**
Fasting BG, mg/dL	207.9 ± 83.0	233.8 ± 111.4	26.8 ± 108	0.5064	219.2 ± 91.3	181.0 ± 75.6	−21.7 ± 103	0.2953	225.7 ± 95.7	160.9 ± 72.2	−67.2 ± 127	0.0949
Total Cholesterol, mg/dL	200.0 ± 31.0	177.4 ± 44.4	−17.9 ± 36	0.1034	183.4 ± 41.2	172.4 ± 43.6	−7.2 ± 30.4	0.0767	187.0 ± 41.4	170.5 ± 34.6	−16.5 ± 50	0.0744
LDL, mg/dL	135.1 ± 28.6	119.3 ± 44.4	−11.2 ± 36	0.2925	119.0 ± 38.4	109.4 ± 44.3	−5.2 ± 26.2	0.1427	123.8 ± 39.4	107.0 ± 31.3	−16.7 ± 39	**0.0259**
HDL, mg/dL	54.3 ± 13.0	43.4 ± 11.4	−8.3 ± 10.6	**0.0155**	51.7 ± 14.2	48.3 ± 12.6	−3.9 ± 8.2	**0.0007**	53.1 ± 18.0	50.5 ± 13.8	−2.1 ± 9.1	0.2163
Triglyceride, mg/dL	107.1 ± 42.0	114.2 ± 38.5	6.7 ± 37.1	0.5430	108.6 ± 64.8	100.3 ± 50.5	−6.3 ± 37.8	0.2349	116.5 ± 57.2	84.0 ± 44.0	−24.1 ± 52	**0.0216**

Numbers are presented as mean ± SD. Abbreviations: BMI: body mass index; TDD: total daily dose; ALDL: low-density lipoprotein; HDL: high-density lipoprotein; SD: standard deviation. Bold *p*-values are significant at α < 0.05.

**Table 4:** Efficacy outcomes at 9-12 and 12-18 months (Participants’ parameters at baseline and after using GLP-1RA).

Parameters	9–12 Months (*n* = 37)				12–18 Months (*n* = 38)			
	Baseline	Follow-Up	MD	*p*-Value	Baseline	Follow-Up	MD	*p*-Value
Weight, kg	90.1 ± 18.7	84.9 ± 20.6	−5.6 ± 6.7	**<0.0001**	91.5 ± 13.9	87.4 ± 15.2	−5.2 ± 6.3	**<0.0001**
BMI, kg/m^2^	34.2 ± 6.8	32.2 ± 7.8	−2.1 ± 2.3	**<0.0001**	34.2 ± 3.7	32.1 ± 4.1	−1.9 ± 2.3	**<0.0001**
TDD of insulin, units/day	84.6 ± 39.4	82.0 ± 41.6	−3.7 ± 24	0.3706	87.3 ± 32.7	81.6 ± 37.2	−5.8 ± 25.7	0.1807
Basal Insulin	35.3 ± 15.8	33.0 ± 15.4	−2.2 ± 8.6	0.1303	36.1 ± 13.5	32.0 ± 15.3	−4.1 ± 9.2	**0.0101**
Prandial insulin	49.3 ± 27.1	48.5 ± 28.6	−1.5 ± 19	0.6418	51.2 ± 23.6	49.6 ± 25.1	−1.7 ± 22.6	0.6567
TDD of insulin, units/kg/day	0.9 ± 0.4	0.9 ± 0.4	−0.0 ± 0.3	0.4534	0.9 ± 0.3	0.8 ± 0.4	−0.1 ± 0.3	0.1511
Time in Range (TIR), %	50.0 ± 11.3	55.6 ± 25.6	17.5 ± 14	0.3440	60.0 ± 0.0	44.5 ± 2.6	---	---
Time Above Range (TAR), %	45.0 ± 18.4	33.4 ± 24.8	−15.0 ± 11	0.3119	37.0 ± 0.0	43.8 ± 12.4	---	---
Time Below Range (TBR), %	4.5 ± 6.4	3.6 ± 3.7	−1.5 ± 3.5	0.6560	3.0 ± 0.0	6.3 ± 7.4	---	---
Vital signs								
Systolic BP, mmHg	126.9 ± 10.8	123.6 ± 15.8	−3.3 ± 16	0.2388	124.8 ± 14.7	122.0 ± 13.6	−2.4 ± 15.2	0.3676
Diastolic BP, mmHg	72.3 ± 10.2	71.5 ± 13.1	−1.0 ± 14	0.6779	71.8 ± 10.8	71.6 ± 10.2	−0.4 ± 12.3	0.8418
Laboratory values								
HbA1c, %	8.7 ± 1.4	8.2 ± 1.7	−0.5 ± 1.2	**0.0210**	8.6 ± 1.4	8.1 ± 1.5	−0.5 ± 0.8	**0.0022**
Fasting BG, mg/dL	196.1 ± 86.0	171.0 ± 54.4	−31.1 ± 82	0.2378	203.5 ± 85.3	176.3 ± 66.6	−22.4 ± 97	0.3561
Total Cholesterol, mg/dL	189.2 ± 42.3	179.7 ± 39.7	−5.8 ± 27	0.3146	188.4 ± 37.4	177.6 ± 38.2	−7.9 ± 34.8	0.2744
LDL, mg/dL	124.8 ± 38.4	111.0 ± 30.3	−14.5 ± 40	0.0960	127.2 ± 39.1	116.7 ± 40.2	−9.9 ± 35.7	0.1858
HDL, mg/dL	53.1 ± 14.7	53.5 ± 20.0	0.3 ± 9.5	0.8699	48.1 ± 10.9	45.0 ± 10.0	−1.8 ± 5.2	0.0972
Triglyceride, mg/dL	103.1 ± 46.9	101.4 ± 65.4	−1.6 ± 43	0.8642	109.5 ± 52.9	102.9 ± 51.9	−3.8 ± 62.6	0.7757

Numbers are presented as mean ± SD. Abbreviations: BMI: body mass index; TDD: total daily dose; ALDL: low-density lipoprotein; HDL: high-density lipoprotein; SD: standard deviation. Bold *p*-values are significant at α < 0.05.

**Table 5:** Safety outcomes while using GLP-1RA.

Variable	Number of Events	Number of Patients
Minor hypoglycemic event		
Within 3 months	4	3/31 (9.7)
Within 3–6 months	19	7/74 (9.5)
Within 6–9 months	9	4/54 (7.4)
Within 9–12 months	16	6/37 (16.2)
Within 12–18 months	7	2/38 (5.2)
Total *	55	18/144 (12.5)
Major hypoglycemic event	1	1 (0.7)
Gastrointestinal events	--	12 (8.3)
Diabetic ketoacidosis event	--	6 (4.2)
Volume depletion or dehydration	--	1 (0.7)
Hospitalization or ED visit during therapy for any reason	28	21 (14.6)

Numbers are presented as frequency or frequency (%). Minor hypoglycemic event: event that did not require ED visit or hospitalization. Major hypoglycemic event: events that required ED visit or hospitalization. Gastrointestinal events: nausea, vomiting, or diarrhea. * This is all minor hypoglycemic events reported by patients during the 18-month follow-up. Abbreviations: GLP-1RA: glucagon-like peptide-1 receptor agonist; ED: emergency department.

## Data Availability

The datasets used and/or analyzed during the current study are available from the corresponding author upon reasonable request.
